# Molecular identification of the chitinase genes in *Plasmodium relictum*

**DOI:** 10.1186/1475-2875-13-239

**Published:** 2014-06-18

**Authors:** Luz Garcia-Longoria, Olof Hellgren, Staffan Bensch

**Affiliations:** 1 Departamento de Biología Animal, Universidad de Extremadura, E-06071 Badajoz, Spain; 2 Department of Biology, Molecular Ecology and Evolution Lab, Ecology Building, Lund University, SE- 22362 Lund, Sweden

**Keywords:** Avian malaria, Chitinase, *Plasmodium relictum*, SGS1, GRW4

## Abstract

**Background:**

Malaria parasites need to synthesize chitinase in order to go through the peritrophic membrane, which is created around the mosquito midgut, to complete its life cycle. In mammalian malaria species, the chitinase gene comprises either a large or a short copy. In the avian malaria parasites *Plasmodium gallinaceum* both copies are present, suggesting that a gene duplication in the ancestor to these extant species preceded the loss of either the long or the short copy in *Plasmodium* parasites of mammals. *Plasmodium gallinaceum* is not the most widespread and harmful parasite of birds. This study is the first to search for and identify the chitinase gene in one of the most prevalent avian malaria parasites, *Plasmodium relictum*.

**Methods:**

Both copies of *P. gallinaceum* chitinase were used as reference sequences for primer design. Different sequences of *Plasmodium* spp. were used to build the phylogenetic tree of chitinase gene.

**Results:**

The gene encoding for chitinase was identified in isolates of two mitochondrial lineages of *P. relictum* (SGS1 and GRW4). The chitinase found in these two lineages consists both of the long (*PrCHT1*) and the short (*PrCHT2*) copy. The genetic differences found in the long copy of the chitinase gene between SGS1 and GRW4 were higher than the difference observed for the cytochrome b gene.

**Conclusion:**

The identification of both copies in *P. relictum* sheds light on the phylogenetic relationship of the chitinase gene in the genus *Plasmodium*. Due to its high variability, the chitinase gene could be used to study the genetic population structure in isolates from different host species and geographic regions.

## Background

Malaria parasites have a complicated life cycle that requires several unique adaptive mechanisms that enable the parasite to successfully invade a variety of different tissues both in the vertebrate host and in the arthropod vector. Presumably as a protection against pathogens, arthropods develop a protective peritrophic membrane (PM) around their midgut after each blood meal which remains for 24 hours and then disappears [1]. The PM acts as a barrier blocking the penetration of parasites and not allowing them to spread to other organs [2]. Parasites in turn, have developed three different ways to overcome this barrier by (i) leaving the erythrocytes before the formation of the PM (as is the case *Wuchereria infection*) [3], (ii) persisting until the PM disappears (e.g. *Leishmania*) [4], or (iii) penetrating the PM (e.g. malaria parasites) [1]. The mechanism which allows malaria parasites to go through the PM of mosquitoes is well described [5-7]. These studies have shown that following the sexual process that takes place in the mosquito stomach, the ookinete has the ability to cross the PM by secreting a chitinase with characteristics of the family 18-glycohydrolases that have catalytic and substrate-binding sites that breaks down this layer [8-10]. After crossing the PM, ookinetes finally transform into oocysts which after maturing (9–11 days [11]) releases the sporozoites that move to the salivary glands where they are ready for infecting a new host (e.g. birds). Therefore, chitinase secretion has an essential role in the completion of the life cycle of malaria parasites.

The mammalian *Plasmodium* parasite species have a single copy of the chitinase gene but with two different structures. In the human and primate malaria parasites, *Plasmodium vivax* and *Plasmodium knowlesi* and the rodent parasites (*Plasmodium berghei*, *Plasmodium yoelii* and *Plasmodium chabaudi*) the chitinase gene is longer and contains both a catalytic domain and a chitin-binding domain; in contrast, the shorter version present in *Plasmodium falciparum* and *Plasmodium reichenowi* lacks the chitin-binding domain [12]. Remarkably, the chicken parasite *Plasmodium gallinaceum* has functional copies of both the long (*PgCHT1*) and the short (*PgCHT2*) chitinase gene [12] suggesting that it is a common ancestor of the mammalian *Plasmodium* parasites that subsequently lost either the short or the long copy of the chitinase gene [13]. The phylogenetic relationships among *Plasmodium* parasites infecting mammals and birds have been intensively debated over the past decades. Some studies have found support for that *P. falciparum* is more closely related to bird parasites than to the other mammalian malaria parasites [13,14], whereas other studies support that the mammalian parasites are forming a monophyletic clade [15,16]. Because *P. gallinaceum* so far is the only bird malaria parasite investigated for its chitinase genes, it is too early to establish that the occurrence of both chitinase copies is representative for bird malaria parasites in general.

*Plasmodium gallinaceum* has been the primary model for studies related with chitinase function in avian malaria [1,17]. However, this species is not the most common malaria parasite in birds. In fact, species belonging to the genus *Plasmodium* show distinct differences in their distribution and prevalence [18]. The most widespread and harmful avian malaria species is *Plasmodium relictum,* found to infect more than 70 different bird species, whereas *P. gallinaceum* has been found to infect only 4 (MalAvi data base 2013-12-02 [19]). *Plasmodium relictum* is one of the most generalist malaria parasite in birds and has several mitochondrial cytochrome b lineages (e.g. SGS1, GRW4, GRW11, LZFUS01) that can be found in almost all continents (MalAvi data base 2013-12-02 [19]). Full understanding of the genetic mechanisms of the infection cycle could help to gain insights into why some parasites are specialist whereas others can infect a large number of different host species.

Despite the wide distribution and harmfulness of *P. relictum,* no study has tried to determine either if this species has the chitinase gene, nor the number of copies it possesses. Therefore, the objectives of this study were (1) to determine whether the two most widespread lineages of *P. relictum* (SGS1 and GRW4) have the gene encoding for chitinase, (2) if these lineages have both copies (CHT1 and CHT2) and (3) finally determine the genetic variability of chitinase genes between the lineages SGS1 and GRW4.

## Methods

### Chitinase identification and sequencing

**Figure 1:**
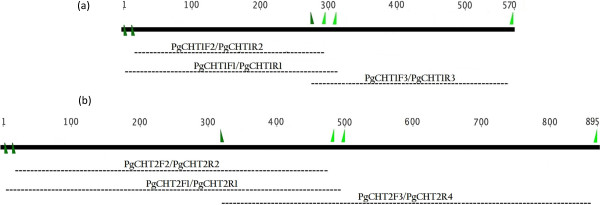
**Primer sites (Forward: dark green arrows, Reverse: light green arrows) along the *cht1 *(a) and *cht2 *(b).** Amplified fragments are represented by dashed lines.

Geneious 6.1. software primer design tool was used to create primers for amplification of overlapping partial regions within the catalytic domain of both copies of the *P. gallinaceum* chitinase genes (long: AF064079; short: AY842482). Each of the copies of the chitinase gene was first aligned to the sequences of all other available mammalian *Plasmodium* parasites in order to identify conserved regions. Figure 1 shows the position of the primers in both fragments of *P. gallinaceum* and the primer sequences are given in Table 1.

**Table 1:** Annealing temperature for all the primers used

**Primer**		**Seq (as ordered)**	**Annealing temp. (°C)**
PgCHT1_F	Forward	5’-ATGATAGAAAATCACCAAGACAAATTTTAGA-3′	50
PgCHT1_R	Reverse	5’-GGTTCCCAGTCAATATCTACACCA-3′	50
PgCHT1_F2	Forward	5’-TAGAGGAATACAAAAGAAGGAAACAAGG −3′	50
PgCHT1_R2	Reverse	5’-CAGTCAATATCTACACCATCTAAATCA −3′	50
PgCHT2_F	Forward	5’-ATTCAAGGTTATTATCCATCATGGGT-3′	53
PgCHT2_R	Reverse	5’-GAAATCCTATACAGCTCAAAGCTCC −3′	53
PgCHT2_F2	Forward	5’-GGGTGTCATATAATCATAATATGAAAGA −3′	53
PgCHT2_R2	Reverse	5’-GACATTGATATTAATTTATCCTCACACA −3′	53
PgCHT1_F3	Forward	5’-AATGACTTTGATTTAGATGGTGTAGAT-3′	55
PgCHT1_R3	Reverse	5’-TAATTGTTCTTTCATAAATAAATGCCA −3′	55
PgCHT2_F3	Forward	5’-ATGAACCCAATGGATCGTTTGATG −3′	58
PgCHT2_R4	Reverse	5’-TAAATTATTAGACAAAGACCACAATCC −3′	58

Two samples from previous experimental infections with *P. relictum*, the cytochrome b lineage SGS1 from crossbills [20] and GRW04 from great reed warblers [21], were used as DNA template. Total genomic DNA from the avian blood samples was extracted by standard ammonium acetate protocol [22]. All samples were screened for chitinase using a nested PCR method for chitinase genes with primers as in Table 1. For both steps, PCR reactions were set up in total volumes of 25 μl, containing 15.4 μl of ddH2O, 1.5 μl of MgCl2 (25 mM), 2.5 μl dNTP (10 mM), 2.5 μl 10x Buffer, 1 μl of each primer (10 μM), 0.1 of Taq polymerase and 1 μl of each sample (25 ng DNA/μl). The PCR temperature profile was 95°C for 2 min followed by 25 or 35 cycles of 95°C for 30 sec, annealing temperature according to Table 1 for 30 sec and 72°C for 30 sec and terminated by a step of 72°C for 10 min. For the SGS1 isolate we used an additional set of primers (PgCHT1_F3, PgCHT1_R3, PgCHT2_F3, PgCHT2R4) to amplify a region 3’ to the fragment obtained with the nested protocol. Positive amplifications were precipitated and sequenced using a dye terminator cycling sequencing (big dye) kit and loaded on an ABI PRISM™ 3100 sequencing robot (Applied Biosystems. Florida. USA).

### Phylogenetic analysis

Sequences from *P. relictum* were aligned with the available chitinase gene sequences from *Plasmodium* spp. (*P. gallinaceum* CHT1: AF064079; CHT2: AY842482; *P. berghei* CHT1: AJ305256; *P. yoelii* CHT1: AB106898; *P. knowlesi* CHT1: XM002257469; *P. vivax* CHT1: AB106896; *P. falciparum* CHT1: AF127445; *P. reichenowi* CHT1: AY842483) using Geneious translation alignment tool. The quality of the alignment was checked by manual inspection. The combined phylogenetic tree for the two copies was constructed in the programme MEGA 5.2 and using a Maximum Likelihood model. Bootstrap values were used in order to obtain a consensus phylogeny using 200 iterations.

## Results

**Figure 2:**
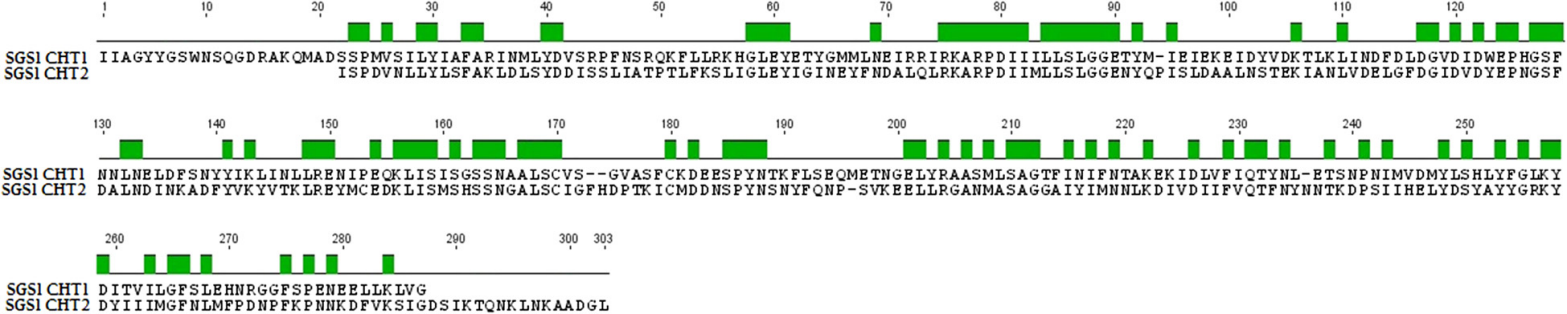
**Overlap of the long (*PrCHT1*) and short copy (*PrCHT2*) sequenced in the mitochondrial lineage SGS1 of *P. relictum*.** The aminoacid sequence is shown in order to illustrate the overlap (green areas).

**Figure 3:**
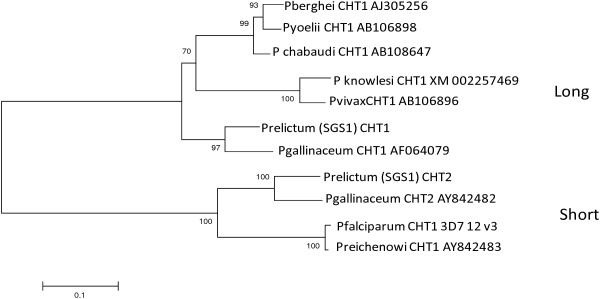
**Maximum likelihood consensus phylogeny using midpoint rooting of the translated CHT1 (long copy) gene and CHT2 (short copy) from 10 different malaria species.** Numbers in branches represent bootstrap values based on 200 iterations.

Both lineages of *P. relictum* (SGS1 and GRW4) had both gene copies encoding for chitinase. The obtained sequences for the lineage SGS1 and GRW4 were 852 bp and 339 bp for the long copy (GenBank accession number KJ452165, KJ452167) and 845 bp and 393 bp for the short copy (GenBank accession number KJ452166, KJ452168). Because the obtained sequences from the GRW4 isolate were short only the data from the SGS1 isolate was used for the phylogenetic analyses. These two regions do not completely overlap (Figure 2). As a result, both sequences were trimmed to only cover the shared sites resulting in a combined alignment covering a region of 802 nucleotides. The phylogenetic analyses found strong support for the separation of the long and the short copies, both being present in the SGS1 isolate of *P. relictum* (Figure 3).

The nucleotide (and amino acid) distances were compared between *P. gallinaceum* and *P. relictum* for both copies using a Pairwise distance matrix. For the long copy a distance of 10.0% was found (9.2%) between SGS1 and *P. gallinaceum*. For the short copy, a distance of 11.0% (10.6%) was found between SGS1 and *P. gallinaceum*. Over the regions for which data of *P. relictum* are available from both isolates of *P. relictum*, SGS1 and GRW4 differed by 1.5% (0.8%) for the short copy and 4.1% (3.6%) for the long copy.

## Discussion

The chitinase gene can consist of one or two copies [9], a long and a short one. Previous studies have established that some malaria parasites only have one copy (e.g. *P. falciparum*[10,23] and *P. berghei*[24]) while only *P. gallinaceum* has both variants [9]. Molecular results showed that *P. relictum* has both copies encoding for chitinase (*PrCHT1* and *PrCHT2*). *Plasmodium relictum* is as far as it is known, the second malaria parasite demonstrated to have both copies. As *P. gallinaceum* and *P. relictum* are quite distantly related among the *Plasmodium* parasites infecting birds [15,16] suggests that the presence of two chitinase gene copies is widespread among the bird *Plasmodium* parasites. Hence, avian malaria parasites are, to date, the only parasites with both copies. Li *et al*. [12] suggested that avian malaria parasites could be the ancestor for the chitinase gene in malaria parasites of primates and rodents. Thus, given the current phylogenetic hypothesis, it can be assumed that mammalian parasites evolved from an avian parasite that carried two copies of the chitinase gene.

The bar-coding gene for molecular identification of *Plasmodium* parasites of birds is the cytochrome b gene [25-27]. When a genetic difference between lineages exceeds 5% this is often followed by distinct morphological differentiation which allows for identification of morphological defined species [26]. Obviously, differences are lower when lineages within the same morphological defined species are compared. The MalAvi data base [19] shows that the genetic variability in the cytochrome b between SGS1 and GRW4 is 1.8% (9 nucleotides different in 480 bp). However, the present study shows that the genetic variability between SGS1 and GRW4 in the chitinase gene was much higher, 4.1% (14 different nucleotides in 339 bp). Moreover, the genetic distance in the cytochrome b between *P. relictum* and *P. gallinaceum* is 6.9% (29 nucleotides different in 480 bp). The results of this study shows that the genetic distance between *P. relictum* and *P. gallinaceu*m in the short copy was 13.1% (44 different nucleotides in 339 bp). Previous studies have identified some nuclear genes with a high variability in *P. relictum*, for instance the msp1 gene [28], that can be used for epidemiological studies of the malaria parasite. In the same way, the chitinase gene could be a good candidate and complement for studies of genetic population structure of the parasites.

In conclusion, the present study demonstrates that the most widespread and harmful avian malaria parasite, *P. relictum*, have the gene encoding for chitinase. In accordance with previous studies on avian malaria (i.e. *P. gallinaceum*), the present study demonstrates that occurrence of both copies (*PrCHT1* and *PrCHT2*) seems to be widespread across avian *Plasmodium* species. Additionally, the present study demonstrates that the genetic variability of the chitinase gene was high between the two analysed lineages of *P. relictum* (SGS1 and GRW4).

To determine the phylogenetic relationship between the chitinase gene in malaria parasites, future studies could search for the number of fragments in other species of haemosporidian parasites and most importantly in the genera closely related to *Plasmodium* that are transmitted by vectors of other dipteran families than Culicidae. Another interesting approach would be to analyse the chitinase gene in parasites isolated from a wide range of bird species with a high prevalence of *P. relictum* and different habitat uses, looking at the gene variability in *P. relictum*.

## Competing interests

The authors declare that they have no competing interests.

## Authors’ contributions

LG carried out the molecular work on the chitinase gene and drafted the manuscript, OH provided the samples analysed. SB designed the study and interpreted the data. OH and SB made important contribution to drafting the manuscript. All authors read and approved the final manuscript.
